# Long-term outcome of the Milano-hyperfractionated accelerated radiotherapy strategy for high-risk medulloblastoma, including the impact of molecular subtype

**DOI:** 10.1093/neuonc/noae189

**Published:** 2024-09-27

**Authors:** Maura Massimino, Francesco Barretta, Chiara Dossena, Simone Minasi, Francesca Romana Buttarelli, Veronica Biassoni, Matilde Oriani, Elisabetta Schiavello, Marica Ficorilli, Olga Nigro, Bianca Pollo, Manila Antonelli, Vittoria Donofrio, Marco Maggioni, Marcel Kool, Emilia Pecori, Sabina Vennarini, Felice Giangaspero, Francesca Gianno, Alessandra Erbetta, Luisa Chiapparini, Roberto Luksch, Elena Barzanò, Cristina Meazza, Marta Podda, Filippo Spreafico, Monica Terenziani, Luca Bergamaschi, Andrea Ferrari, Michela Casanova, Stefano Chiaravalli, Giovanna Gattuso, Piergiorgio Modena, Simon Bailey, Loris De Cecco

**Affiliations:** Pediatric Unit, Fondazione IRCCS Istituto Nazionale dei Tumori, Milano, Italy; Department of Clinical Epidemiology and Trial Organization, Fondazione IRCCS Istituto Nazionale dei Tumori, Milano, Italy; Integrated Biology Platform, Department of Applied Research and Technology Development, Fondazione IRCCS Istituto Nazionale dei Tumori, Milano, Italy; Fondazione IRCCS Istituto Nazionale dei Tumori, Milano, Italy; Fondazione IRCCS Istituto Nazionale dei Tumori, Milano, Italy; Pediatric Unit, Fondazione IRCCS Istituto Nazionale dei Tumori, Milano, Italy; Integrated Biology Platform, Department of Applied Research and Technology Development, Fondazione IRCCS Istituto Nazionale dei Tumori, Milano, Italy; Pediatric Unit, Fondazione IRCCS Istituto Nazionale dei Tumori, Milano, Italy; Integrated Biology Platform, Department of Applied Research and Technology Development, Fondazione IRCCS Istituto Nazionale dei Tumori, Milano, Italy; Pediatric Unit, Fondazione IRCCS Istituto Nazionale dei Tumori, Milano, Italy; Neuropathology Department, Fondazione IRCCS Istituto Neurologico Carlo Besta, Milano, Italy; Department of Radiological, Oncological and Anatomo-Pathological Sciences, La Sapienza University, Rome, Italy; Pathology Department, Ospedale Santobono-Pausilipon, Napoli; Italy; Pathology Department, IRCCS Fondazione Policlinico, Milano, Italy; Hopp Children’s Cancer Center Heidelberg (KiTZ), Heidelberg, Germany; Princess Maxima Center, Amsterdam, Netherlands; Pediatric Radiotherapy Unit, Fondazione IRCCS Istituto Nazionale dei Tumori, Milano, Italy; Pediatric Radiotherapy Unit, Fondazione IRCCS Istituto Nazionale dei Tumori, Milano, Italy; Department of Radiological, Oncological and Anatomo-Pathological Sciences, La Sapienza University, Rome, Italy; Department of Radiological, Oncological and Anatomo-Pathological Sciences, La Sapienza University, Rome, Italy; Neuroradiology Department, Fondazione IRCCS Istituto Neurologico Carlo Besta, Milano, Italy; Neuroradiology Department, Fondazione IRCCS Policlinico S. Matteo, Pavia, Italy; Pediatric Unit, Fondazione IRCCS Istituto Nazionale dei Tumori, Milano, Italy; Pediatric Unit, Fondazione IRCCS Istituto Nazionale dei Tumori, Milano, Italy; Pediatric Unit, Fondazione IRCCS Istituto Nazionale dei Tumori, Milano, Italy; Pediatric Unit, Fondazione IRCCS Istituto Nazionale dei Tumori, Milano, Italy; Pediatric Unit, Fondazione IRCCS Istituto Nazionale dei Tumori, Milano, Italy; Pediatric Unit, Fondazione IRCCS Istituto Nazionale dei Tumori, Milano, Italy; Pediatric Unit, Fondazione IRCCS Istituto Nazionale dei Tumori, Milano, Italy; Pediatric Unit, Fondazione IRCCS Istituto Nazionale dei Tumori, Milano, Italy; Pediatric Unit, Fondazione IRCCS Istituto Nazionale dei Tumori, Milano, Italy; Pediatric Unit, Fondazione IRCCS Istituto Nazionale dei Tumori, Milano, Italy; Pediatric Unit, Fondazione IRCCS Istituto Nazionale dei Tumori, Milano, Italy; Genetics Laboratory, Ospedale S.Anna, Como, Italy; Pediatric Oncology Department, Sir James Spence Institute of Child Health Royal Victoria Infirmary Queen Victoria Road Newcastle upon Tyne, UK; Integrated Biology Platform, Department of Applied Research and Technology Development, Fondazione IRCCS Istituto Nazionale dei Tumori, Milano, Italy

**Keywords:** HR medulloblastoma, HART, SHH subtype

## Abstract

**Background:**

We applied the strategy for M+ medulloblastoma across all high-risk subgroups, including LC/A histology, TP53 mutations, and MYC/MYCN amplification.

**Methods:**

Patients over 3 years old received, after surgery, staging and histo-biological analysis, sequential high-dose-methotrexate(HD-MTX), high-dose-etoposide(HD-VP16), high-dose-cyclophosphamide(HD-Cyclo), and high-dose-carboplatin(HD-Carbo). Hyperfractionated-accelerated-radiotherapy–craniospinal(HART-CSI), administered twice daily 1.3 Gy-fractions reached a total dose tailored to the patients’ age and pre-radiation response to chemotherapy(CT): 31.2 Gy if under 10-years-old and complete response(CR) or partial response(PR) obtained or absence of metastatic disease, 39 Gy in other/older patients. Boosts to posterior fossa/residual metastatic(M+) deposits were given up to a total dose of 60 Gy/9 Gy, respectively, but avoided if metastatic nodules were very big or patients were very young. Two courses of high-dose-thiotepa were delivered in case of not CR/PR after the pre-radiotherapy (RT) phase and in all M0 patients either—pre/post-HART. Subgrouping was performed where the tissue was available.

**Results:**

Eighty-nine patients were enrolled, with a median age of 8.8 years, and a median follow-up of 136 months. Overall survival (OS) and event-free survival (EFS) at 5/15 years were 75.9/66.5% and 68.2/65.3%, respectively; 5/28 fatal events were not related to relapse(3 developed secondary malignancies). Sex, age less than 10 years, histological subtype, presence of MYC/MYCN amplification, reduction in CSI dose, omission of RT-boosts, implementation of myeloablative therapy, presence–absence of metastases did not impact prognosis.Patients progressing after pre-HART CT(14/89) and stable-disease(SD)+PD after HART(10/89) negatively affected outcome(*P* < .001).Subgrouping in 66/89 patients’ samples demonstrated a significantly worse EFS for patients with Sonic Hedgehog(SHH)-tumors(#15, 2 with constitutional *TP53*-mutations) versus groups 3 and 4(15 and 29 patients, respectively, group3/4 in 7).Patients younger than 10 received lower CSI doses if stratified according to CT response.

**Conclusions:**

This strategy, partly adopted in the ongoing SIOPE protocol, confirmed improved EFS and OS over previously reported outcomes in all high-risk categories; SHH tumors appeared the most aggressive.

**Key Points**

- chemotherapy and radiotherapy responses statistically correlated to prognosis in a high-risk medulloblastoma series.
- a reduction of craniospinal irradiation dose was feasible after optimal response to pre-radiation chemotherapy.
- patients with SHH subtype prognosis medulloblastoma had the worst prognosis.

**Importance of the Study**

We present a series of 89 high-risk medulloblastoma cases treated uniformly over a span of more than 20 years, with a median follow-up exceeding 10 years. Our findings show a relatively high overall survival (OS) and event-free survival (EFS) at 5 and 15 years (75.9%/66.5% and 68.2%/65.3%, respectively). Additionally, our analysis indicates that response to both chemotherapy and radiotherapy—delivered using the hyperfractionated accelerated radiotherapy (HART) schedule—were significant prognostic factors. Notably, achieving an optimal response to chemotherapy allowed us to reduce craniospinal irradiation doses in 31 out of 50 patients under 10 years old at diagnosis without compromising their outcomes. Furthermore, we found that tumors classified under the Sonic Hedgehog (SHH) subtype had the poorest prognosis, regardless of histological variety and the extent of metastases.

In 2009, we published an almost mono-institutional series of 33 patients treated for metastatic medulloblastoma, the median age was 10 years, delineating the so-called “Milan strategy for metastatic medulloblastoma.”^1^

Thereafter we adopted the same treatment strategy in all those patients presenting with newly recognized,^2,3^ poor prognostic factors both for histology and biological alterations, such as large cell/anaplastic (LC/A) morphologic subtypes, *TP53* mutations, and/or MYC and MYCN.^4,5^ This strategy was maintained with some amendments until the approval of the SIOP high-risk medulloblastoma protocol^2^ where, among the 3 randomized arms following induction chemotherapy(CT), one included the use of hyperfractionated accelerated radiotherapy (HART) to deliver craniospinal irradiation (CSI). HART and tailored use of high-dose CT were the basis of the Milano strategy used until 2021.^1^

In this paper, we have updated the results obtained in 89 consecutive patients with high-risk medulloblastoma treated between 1998 and 2021, in addition, analyzing the available tumor material in order to include in this report the same clinical and biological factors that we are using in the screening for inclusion in the ongoing SIOP protocol (EudraCT 2018-004250-17).

## Patients and Methods

### Staging and Treatment

**Figure 1.**
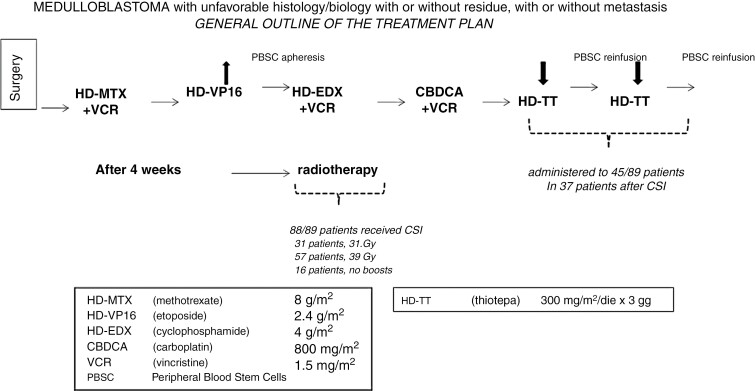
Treatment scheme.

Details of the treatment have been already described in previous papers^1,4,5^ and are reported in **Figure 1**. All consecutive patients diagnosed with metastatic medulloblastoma after 1998, and subsequently also with other high-risk features with or without metastatic disease, underwent tumor resection at different neurosurgery units and were subsequently referred to Fondazione IRCCS Istituto Nazionale dei Tumori, in Milan, for staging and adjuvant treatment. All histological samples were centrally reviewed before adjuvant treatment by F.G., M.A., and F.Gi.. The lower age limit for patients to be treated using this strategy was 3 years, while there was no upper age limit. All patients postoperative staging consisted of whole central nervous system MRI and spinal fluid (CSF) cytology (the CSF was obtained routinely after more than 2 weeks after surgery through a lumbar puncture), and all were treated with adjuvant CT as shown in Figure 1. HART was administered in all cases, in 2 daily 1.3 Gy fractions 6 hours apart, for 5 days a week, reaching a total dose of CSI tailored to the patient’s age at diagnosis and pre-radiation response to CT. CSI treatment was delivered at a total dose of 31.2 Gy in children under 10 years old if at least a partial remission (PR) was obtained and at 39 Gy in older patients. (PR was defined as ≥50% decrease [compared with baseline] in the sum of the products of perpendicular diameters of all measurable lesions and CSF negative if previously negative [positive or negative if previously positive]) The posterior fossa boost was given in 1.3 or 1.5 Gy fractions twice a day to obtain a total dose of 59.7 and 60 Gy in patients younger and older than 10 years old, respectively. For residual nodules in the posterior fossa or metastatic deposits, after CSI, an additional boost of 9 Gy was delivered in 6 twice-daily 1.5 Gy fractions. The boosts to the posterior fossa or to metastatic deposits were avoided in particular cases ie, those patients with huge metastatic nodules where the primary tumor could be even smaller than metastatic sites, or in very young patients where the risk of acute and late effects resulted in more personalized radiotherapy (RT) schedules.

After RT, patients were prescribed 2 courses of thiotepa at myeloablative doses (300 mg/m^2^/day for 3 days) followed by autologous stem cell rescue if complete remission (CR) had not been achieved before CSI. Since 2013, due to the reports of severe toxicities in younger patients when submitted to a similar regimen in other institutions,^6,7^ we delivered the courses of high-dose thiotepa before HART. We did not observe the same level of toxicity as reported by other authors.^6,7^ However, since those toxicities predominantly affected patients younger than 10 years, we did not administer high-dose thiotepa to patients in that age group after irradiation in 2013. Additionally, we refrained from this treatment if patients had achieved at least stable disease after pre-radiation CT.

Patients with LC/A medulloblastoma, MYC, MYCN amplification, and *TP53* mutation, either somatic or germ-line, or any combination of these factors, received the myeloablative courses before or after CSI according to the same schedule. Peripheral blood stem cell collection involved leukapheresis and cryopreservation after administering etoposide or cyclophosphamide during the pre-radiation phase of the treatment.

Patients not receiving the 2 myeloablative courses were submitted to vincristine and lomustine maintenance for one year after HART.

Morphological subgrouping is described in Supplementary Table 1 and Figure 1A-C.

### Methylation Profile

To analyze the methylation profile of the high-risk medulloblastoma patients, genomic DNA was extracted from FFPE samples with DNA Blood & Tissue Kit (cod. 69504, Qiagen) following the manufacturer’s instructions. The concentration and quality of the DNA were assessed respectively with the Qubit 3.0 fluorometer (Life Technologies) and the TapeStation 4200 system (Agilent Technologies). In total, 500 ng of DNA was processed for methylation analysis using Illumina Infinium Methylation EPIC v2.0 Kit (Illumina). Briefly, gDNA was bisulfite converted on a 96-well plate using the EZ DNA Methylation Kit (Zymo Research) according to the manufacturer’s instructions followed by a restoration step using the Infinium FFPE DNA Restore Kit (Illumina). Denaturation, amplification, and ligation were carried out following the manufacturer’s instructions. DNA was purified with ZR-96 DNA Clean & Concentrator-5 kit (Zymo Research) and eluted in 10 µL of DiH2O. After amplification, fragmentation, precipitation, and resuspension, DNA was hybridized on BeadChip. Finally, chips were scanned with the iScan (Illumina). Image processing was performed by the Illumina iScan System and IDAT files containing intensity data were extracted.

For data Quality Controls (QCs) GenomeStudio Software (v 2011.1) was used. IDAT files and Manifest file containing information about each CpG site, including the gene name and location relative to the CpG islands were uploaded^8^ in order to convert IDATs to β-value, which allows the determination of the methylation status for each CpG site within each sample. Next, the number of CpG sites detected was checked to have more than 779.000 CpG sites (for FFPE/degraded samples). Finally, we examined the correct bisulfite conversion and the specificity of the probes (Infinium I and Infinium II) in each sample.

After QCs, IDATs were processed with a DNA methylation-based classifier of central nervous system tumors on www.molecularneuropathology.org site.^9^ Brain_classifier_v12.8_sample_report workflow was executed and copy number variants profile, classification, and MGMT promoter methylation were predicted.

### Statistical Analysis

Descriptive statistics of the demographic and clinical-pathological characteristics of the patients were reported. Two survival outcomes were evaluated: overall survival (OS) and event-free survival (EFS). OS was defined as the time elapsed between the date of study enrollment to death due to any cause. EFS was defined as the time elapsed between the date of study enrollment and local recurrence, distant metastasis, or death due to any cause, whichever came first. Time was censored for patients still alive and event-free at the end of the follow-up.

The OS and EFS curves were estimated using the Kaplan–Meier method and compared by means of the log-rank test. Association between the outcomes and sex (male, female), age (<10, ≥10), post-surgical residues (yes, no), histological subtype (classic, LC/A, N/D), molecular subgroup (Sonic Hedgehog (SHH), group 3, group 4, non-WNT/non-SHH ie, group 3 or group 4), MYC/MYCN amplification (present, absent), CSI dose reduction (yes, no), omission of boosts (yes, no), intensification with myeloablative courses (yes, no), the extent of metastases at diagnosis (M0, M1-M3),^10^ sites of relapse (local, distant, or combined relapse), response to pre-HART CT (CR/PR/SD, PD) and after HART (CR/PR, SD/PD), and overall response to pre-HART and after HART (no: if CT response was PD or RT response was SD or PD, yes: if not already classified as “no” and CT response was SD, PR, or CR or RT response was PR or CR) were evaluated. The median follow-up was estimated with the reverse Kaplan–Meier method^11^ on the OS data. Subgroup analyses were performed in patients for which molecular subgrouping was available. The median time to relapse was estimated as the median observed relapse time between relapsed patients only.

Four groups of association tests were performed by means of the Fisher’s Exact test: age and MYC/MYCN amplification, histological subtypes, molecular subgroups, response to pre-HART CT and after HART, and RT boost; site of relapse and histologic subtype, CSI dose reduction, intensification with myeloablative courses, molecular subgroups, and RT boost; molecular subgroup and metastases, histological subtype, and MYC/MYCN amplification; metastases and overall response to pre-HART and after HART.

Multivariable Cox models were performed for the 2 outcomes including the following putative prognostic covariates: age, molecular subgroups, response to pre-HART CT, response after HART, and metastases. In the Cox models, the categorical variables were modeled using dummy variables, and the continuous variables were modeled using 3-knot restricted cubic splines.^12^

The statistical analyses were conducted using SAS software (SAS Institute) and R software (http://www.r-project.org/).

This retrospective research received the Independent Ethical Board approval on July 26, 2023, as protocol 174/23.

Appropriate information and consent forms for patients, parents, and tutors were signed.

## Results

Eighty-nine patients were treated according to uniform guidelines from February 1998 to September 2021, with a median follow-up of 136 months (first and third quartile: 75–238 months) at the time of this report. The median age at diagnosis was 8.8 years (range 3–32 years), with 64 males (M/F ratio: 2.56). Fifty patients were younger than 10 years at diagnosis and 43 had residual disease at the primary site after surgery. Metastatic disease was evaluated as M1 in 11, M2 in 17, and M3 in 49 while 12 patients had other high-risk features (LC/A histology in 9, LC/A histology and MYC amplification in 1, and TP53 mutation in the remaining 2 patients) and no metastatic disease (M0). Histological medulloblastoma subtype was not able to be determined in one case, was classic in 57, large-cell/anaplastic (LC/A) in 24, and nodular/desmoplastic in the remaining 7. MYC and MYCN amplification were evaluated in 74/89 patients and were found in 17 and 4, respectively (Table 1).

**Table 1.** Clinical-Pathological and Disease Characteristics of the 89 Medulloblastoma Patients

	*n* (%)
Age, years	
<10	50 (56.2)
≥10	39 (43.8)
Sex	
Male	64 (71.9)
Female	25 (28.1)
Histology	
Classic	57 (64.0)
Large cell/anaplastic	24 (27.0)
Desmoplastic/nodular	7 (7.9)
Undetermined	1 (1.1)
Molecular subgrouping	
Sonic hedgehog	15 (22.7)
Group 3	15 (22.7)
Group 4	29 (43.9)
Non-WNT/Non-SHH	7 (10.6)
MYC amplification	
Yes	17 (23.0)
No	57 (77.0)
MYCN amplification	
Yes	4 (5.4)
No	70 (94.6)
Extent of metastases	
M0	12 (13.5)
M1	11 (12.4)
M2	17 (19.1)
M3	49 (55.1)
Intensification with myeloablative courses	
Yes	45 (50.6)
No	44 (49.4)
Chemotherapy response	
Complete response	23 (29.5)
Partial response	32 (41.0)
Stable disease	9 (11.5)
Progressive disease	14 (17.9)
Radiotherapy response	
Complete response	32 (58.2)
Partial response	13 (23.6)
Stable disease	9 (16.4)
Progressive disease	1 (1.8)
Response to chemotherapy and radiotherapy	
Yes	60 (76.9)
No	18 (23.1)
Cranio-spinal irradiation dose	
39 Gy	57 (64.8)
31.2 Gy	31 (35.2)
Radiotherapy boost	
Yes	72 (81.8)
No	16 (18.2)
Post-surgical status	
Evidence of disease	43 (48.9)
No evidence of disease	45 (51.1)
Site of relapse	
Local relapse	8 (30.8)
Distant metastasis	16 (61.5)
Both	2 (7.7)

Of the 24 patients with LC/A histology, 15 had also metastatic disease, 7 had MYC/MYCN amplification, and 12 had none of the 2.

**Figure 2.**
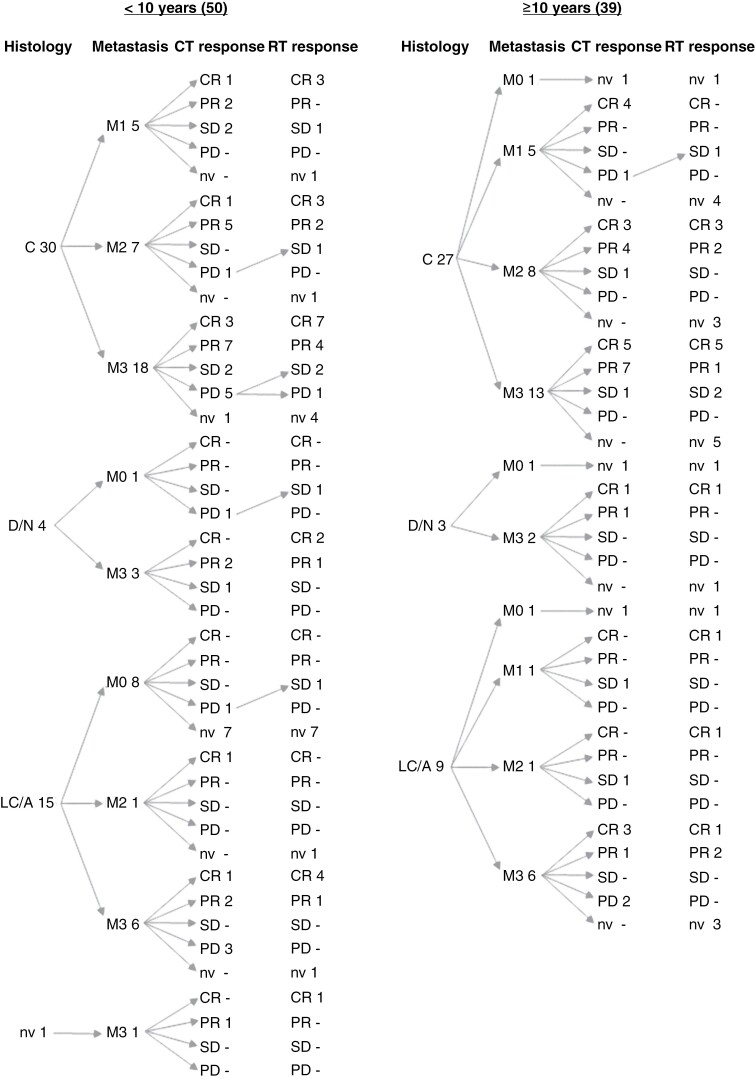
Patients’ description according to age, histologic subtype, metastatic status, and response to chemotherapy and radiotherapy.

Molecular subgrouping was evaluated in 66 retrospective tumor samples and was described as SHH in 15, group 3 in 15, and group 4 in 29, either group 3 or 4 in 7, while the WNT subgroup was not found in any sample. Supplementary Figure 2 reports the *Oncoplot* of these 66 patients. The immunohistochemistry (IHC) results for the SHH molecular subgroup were consistently concordant with the methylation profile and we could identify group 3/group 4 cases through IHC. However, in 23 cases, we were unable to perform the methylation profiling due to either material retrieval issues or inadequate samples. Of the SHH tumors, 4/13 evaluable samples showed MYC or MYCN amplification that was present also in 6/13 group 3 and 8/27 group 4 available tumors. Among the 15 samples belonging to the SHH subgroup, there were 6 with LC/A histotype (1 with MYCN amplification). Response to pre-radiation CT was evaluable in 78 patients, one patient died due to ventricular shunt sepsis soon after treatment commenced 10 were without macroscopic disease after surgery and had no evidence of metastatic disease: 23 had obtained a CR, 32 had a PR, 9 had SD while tumor progressed in 14 patients. The 14 patients progressing after pre-radiation CT proceeded to radiation therapy according to the HART schedule obtaining a CR in 4, a PR in 4, an SD in 5, and 1 patient had further progressive disease. Only one patient, however, also submitted to high-dose thiotepa after HART obtained CR, and was alive at 144 months after diagnosis. Response to RT in the 55 patients with evaluable disease was CR in 32, PR in 13, SD in 9, and PD in 1. Figure 2 shows a scheme of the responses to pre-radiation CT and RT according to patient age, tumor histology, and extent of metastases.

Radiation boosts either on posterior fossa or on residual metastatic sites were not adopted in 16 cases while myeloablative courses were implemented in 45 patients, 37 of which were after CSI. Thirty-one of the 50 patients younger than 10 years at diagnosis received lower CSI doses.

**Figure 3.**
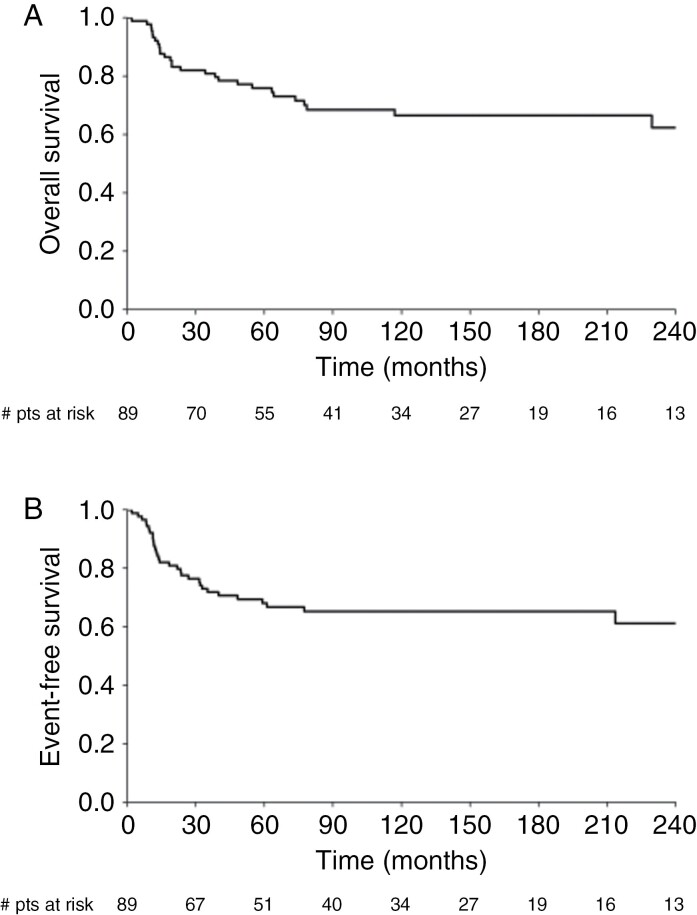
Kaplan–Meier curves for overall survival (A) and disease-free survival (B) in the whole cohort.

**Figure 4.**
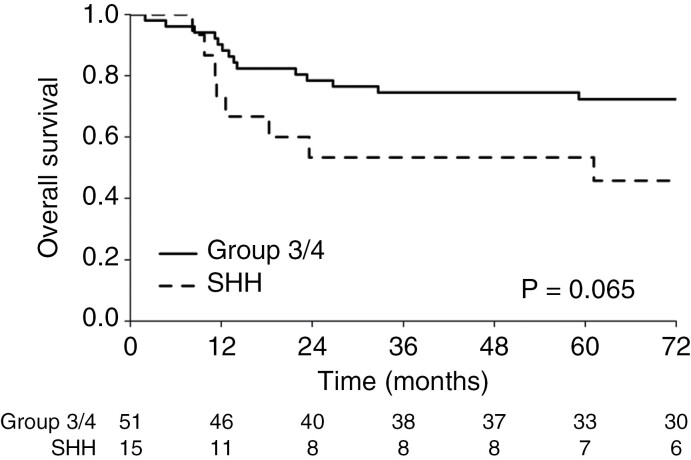
Kaplan–Meier curves for overall survival according to a molecular subgroup in the 66 patients with available classification.

OS and EFS at 5 and 15 years were 75.9/66.5% and 68.2/65.3%, respectively (Figure 3). Five of the twenty-eight fatal events were not associated with medulloblastoma progression or relapse (3 patients died of secondary tumors- one glioblastoma, one meningeal sarcoma, one acute myeloid leukemia, one due to shunt infection, and the last one for uncontrolled seizures). Sex, age below 10 years, post-surgical residual disease, histological subtype, MYC and MYCN amplification, CSI dose reduction, omission of boosts, intensification with myeloablative courses, and presence or absence of metastatic disease did not impact prognosis. Patients progressing after pre-HART CT (#14) and SD + PD after HART (# 10) did significantly worse (*P* < .001) with one and zero survivors, respectively. Subgrouping was performed in 66/89 patients and EFS showed a trend toward a poorer outcome for the 15 patients with SHH-tumors (2 with constitutional *TP53*-mutations) versus patients with group 3 and 4 (#51) medulloblastoma (15-year EFS: 45.7%, 95% CI: 26.1%–80.2% vs 69.8%, 58.1%–83.9% CI: *P* = .065; Figure 4). The poor outlook of SHH tumor patients was associated with a lack of overall response (5/5 non-respondent patients died within 2 years vs 15-year EFS: 72.9, 95% CI: 46.8%–100% for the other 10; *P* < .001) but no to the extent of metastases nor to LC/A histology.

Age ≥ or < 10 years at diagnosis was not associated with MYC and MYCN amplification and with histological subtypes, but patients <10 years had more group 3 tumors (38% vs 11% in patients ≥10 years, *P* = .067). Patients younger than 10 years had a worse response to CT (26% vs 8% in patients ≥10 years, *P* = .073) and received less RT boosts (27% vs 8% in patients ≥10 years, *P* = .027).

Of the 26 patients that suffered a relapse, the median time to relapse was 13.9 months from diagnosis; 8 had only local relapse, 2 had local and distant and the remaining 16 had only distant relapse. The site of relapse was not associated with histologic subtype, CSI dose, or implementation of myeloablative courses. Of the 8 patients only relapsing locally (the other 2 had also distant relapse) 3 had not received the boost but there were no differences in site of relapse according to molecular subgroups and delivery of RT boosts.

Patients with SHH tumors had significantly less M1 metastatic disease than those with Group 3 plus Group 4 patients (0% vs 20%, *P* = .063), and more LC/A subtype tumors (40% vs 18%, *P* = .020). MYC/MYCN amplification was not associated with molecular subgrouping.

Considering the response to CT and RT, we described 12 patients who had either no positive response to the first or to the second treatment and 6 who did not respond to both (as shown by the oblique arrows in Figure 2). This clinical behavior was associated with non-metastatic disease (M0 vs M1/M2/M3; 89% vs 100%, *P* = .051).

In a multivariable analyses (Table 2) model including patients’ age, molecular subgrouping, extent of metastases, and response to CT and RT, EFS was associated with both CT (*P* < .001) and RT responses (*P* = .015, respectively), while for OS only response to CT was significant (*P* < .001).

**Table 2.** Results of the Multivariable Fine and Gray Models for Overall Survival and Event-Free Survival

	Overall survival		Event-free survival	
	HR (95% CI)	*P*	HR (95% CI)	*P*
Age		.948		.505
13.7 vs 6.2	0.88 (0.30–2.54)		0.60 (0.22–1.70)	
Molecular subgrouping		.197		.100
Group 3 vs SHH	0.44 (0.08–2.36)		0.20 (0.04–0.88)	
Group 4 vs SHH	1.86 (0.43–7.95)		0.47 (0.11–1.98)	
Chemotherapy response		<.001		<.001
Poor vs Good	22.9 (4.2–125.4)		20.9 (3.92–111.3)	
Radiotherapy response		.197		.015
Poor vs Good	2.69 (0.60–12.03)		5.60 (1.39–22.46)	
Extent of metastases		.343		.770
M1-M3 vs M0	4.60 (0.20–108.1)		0.67 (0.04–10.10)	

*Abbreviations:* HR, hazard ratio; CI, confidence interval; p, Gray test *P*-value; SHH, sonic hedgehog.

Twelve patients developed secondary tumors; 3 with a fatal outcome as already described. The other 9 survived, despite having more than one occurrence in any single patient (papillary thyroid carcinoma in 5, meningiomas in 3, eccrine poroma in one, endometrial polyposis in one, melanocytic tumor of uncertain malignant potential (MELTUMP), atypical Spitz nevus and melanoma in one each, liver adenomatosis in 2, basal cell carcinoma in 2, ovarian adenoma in one).

A systematic neurocognitive prospective evaluation of the patients was not performed; nevertheless, Supplementary Table 2 reports surviving patients’ outcomes according to age at diagnosis, posterior fossa syndrome suffered after surgery, CSI doses and posterior boosts received, high-dose thiotepa implementation, and months of follow-up. All 54 out of 58 tested patients who remained in complete remission (CR), regardless of the total CSI dose, additional boosts, or high-dose chemotherapy received, exhibited at least one endocrine alteration. Of the surviving patients, 23 out of 34 who received a total CSI dose of 39 Gy had achieved a degree, employment, or social integration, compared to 16 out of 23 who received 31.2 Gy (*P* = n.s); hearing impairment was observed in 14 out of 27 patients from the first group and 12 out of 16 from the second group (*P* = n.s.). Posterior fossa syndrome after surgery occurred in 8 out of 35 patients in the group that received 39 Gy, while none of the 23 patients in the group that received 31.2 Gy experienced this syndrome (*P* = .017).

## Discussion

During the last 20 years, different cooperative groups and single institutions have tried to improve the prognosis of high-risk medulloblastoma by using different therapeutic approaches, ie, higher CSI doses^13^ with or without concomitant drugs,^14,15^ myeloablative CT courses,^1,16,17^ or altered RT schedules,^18^ or any sum of these different strategies including intrathecal CT.^19^

In addition, with advances in molecular diagnostics and more detailed analysis, the definition of high-risk medulloblastoma has evolved over the years and now many protocols, like SIOP HR-MB,^3^ exclude those patients with macroscopic residual disease after surgery^20^ and no other risk factors but include those with biological and clinical high-risk factors.^21,22^

In our experience, we included in the Milano strategy only patients with metastatic disease until 2007, thereafter we included patients whose medulloblastoma had LC/A morphologic subtypes, *TP53* mutation, and/or MYC and MYCN amplifications.^4,23^

Our strategy, initially published in 2009 exclusively for 33 patients with metastatic disease, produced encouraging results in a total of 89 patients (including 33 previously reported), with a 65.3% EFS and 66.5% OS at 15 years. These outcomes were the best reported, despite the absence of detailed biological analysis at the time of the first manuscript. Further analysis on the whole cohort of 89 patients and after a median follow-up of over 10 years provided an opportunity to reevaluate the results, now including both biological and clinical risk factors. Retrospective analysis demonstrated that when treated on the Milano protocol many reported factors such as histological subtypes, MYC/MYCN amplification, metastatic status, and post-surgical residual disease had no prognostic impact. No series so far has described similar results with a number of different treatment approaches. The lack of prognostic significance of the various factors identified by other groups in large prospective clinical trials^15^ could be attributed to the therapy nullifying their prognostic value. More likely, however, this discrepancy may be due to our small patient cohort. The Children’s Oncology Group ACNS0332 Study could in fact analyze 261 randomized patients and concluded that therapy intensification with carboplatin improved EFS by 19% at 5 years for children with high-risk group 3 medulloblastoma.^15^

The newly ongoing SIOP trial for high-risk medulloblastoma including one arm using HART as the radiation approach^2^ will reevaluate if these factors maintain their prognostic value.

The series of medulloblastoma reported by Gajjar et al.^23^ included 103 high-risk patients treated with uniform therapy: Post-surgical irradiation was followed by 4 courses of vincristine, cisplatin, and cyclophosphamide at a myeloablative dose.^16^ In their most recent analysis, metastatic disease, LC/A histology, MYC, and MYCN amplification were shown to be adverse prognostic factors although residual disease alone was not. SHH subgroup associated with the above-described variables, GLI2 amplification, and chromosome 17p loss, also had a poorer outcome.^24^

The most important negative prognostic clinical factors in our series were progression after CT given in the pre-radiation phase and the absence of response, or progression, after RT. For some patients, in the first years of this strategy, RT was followed by high-dose thiotepa with the aim of improving the poor prognosis associated with a lack of response to pre-HART CT (10% of patients).^1^ The response to pre-irradiation CT was described as prognostic by several other authors^17,25–27^ and maybe a surrogate marker of biological factors. Moreover, the latest retrospective series reported by the HIT group, involving 84 patients with visible tumors at the completion of planned therapy, found that neither additional chemotherapy (without myeloablative treatment) nor surgery improved patients outcome.^28^ This reinforced the need for an optimal response to the early phases of treatment. The poor prognosis of SHH tumor patients was associated with the response but not to the extent of metastases or LC/A histology. This same observation on SHH outcome, also independent of *TP53* mutation, was reported by the Hospital for Sick Children, Toronto, Canada (Sickkids) group in 2 papers^2,29^ and by the widest randomized study in high-risk medulloblastoma so far reported by the Children’s Oncology Group ACNS0332 Study.^15^

It is worth noting the potential for reducing CSI HART doses in patients under 10 years of age at diagnosis (31/50). This reduction may also reduce their long-term sequelae. Furthermore, omitting posterior fossa and metastatic boosts during RT did not have a negative prognostic impact. The possibility of reducing total doses of CSI and boosts in the subset of high-risk patients was also explored by an SFOP study as reported by Verlooy in 2006^30^ with modulation of supratentorial brain doses and boosts associated with the response obtained by pre-radiation CT. They described also a CSI dose as low as 25 Gy for patients with CR tumors after CT in the pre-radiation phase.

Approximately 20% of long-term survivors experienced the development of secondary tumors varying from basal cell carcinoma to aggressive cancers, resulting in death in 3 cases. Unfortunately, with longer and more thorough follow-ups, the likelihood of detecting secondary tumors increases, as observed even with less intensive treatments.^31^

In conclusion, our findings suggest that certain subgroups of patients, particularly those with SHH tumors and those diagnosed at a younger age (under 10 years), may benefit from intensifying CT or alternative drug regimens in the pre-radiation phase. However, upon achieving an optimal response, these patients could potentially undergo reductions in CSI doses and boosts. In this series, the neurological, sensory, and psychological disabilities, intelligence quotient scores, educational attainments, employment opportunities, and the ability to lead independent lives were highly varied and difficult to correlate directly with the post-surgical treatments received. We now know that toxicities are the sum of many factors: hydrocephalus, surgical maneuvers, posterior fossa syndrome, and thereafter adjuvant treatment delivered, including standard-dose CT, quality of rehabilitation, school and social attendance, sports activities, and socioeconomic status among others.^32–34^ Ongoing protocols for high-risk medulloblastoma, which integrate biological, quality of life, endocrine, and neurocognitive evaluations within a prospective design, aim to elucidate, through phase 3 studies, the most effective strategies for both cure and mitigation of side effects in this historically challenging patient population.^3^

## Supplementary material

Supplementary material is available online at *Neuro-Oncology* (https://academic.oup.com/neuro-oncology).

noae189_suppl_Supplementary_Table_S1

noae189_suppl_Supplementary_Table_S2

noae189_suppl_Supplementary_Figure_S1

noae189_suppl_Supplementary_Figure_S2

noae189_suppl_Supplementary_Material

## Data Availability

The data will be made available upon reasonable request.

## Dedication

Professor Giangaspero has always been a true master, possessing a rare gift for profound insights, yet remaining open to our ideas and the modest contributions we brought as clinicians. Like us, he pursued a deeper understanding of pediatric CNS tumors—not only with the rigorous ambition of a scientist, but also with the unyielding, and sometimes irrational, determination we clinicians share, driven by the hope of finding a cure and bringing healing to all.
