# Supplementary material for: Long-term outcome of the Milano-hyperfractionated accelerated radiotherapy strategy for high-risk medulloblastoma, including the impact of molecular subtype
Source: Neuro Oncol. 2024 Sep 27;27(1):209–18. doi: 10.1093/neuonc/noae189 (PMC11726337; doi:10.1093/neuonc/noae189)
Supplement: noae189_suppl_Supplementary_Table_S1 [file noae189_suppl_supplementary_table_s1.docx]

| **Antibody** | **Manufacturer** | **Host** | **Isotype** | **Clone** | **Dilution** | **Protocol BOND III** | **Epitope Retrival** |
| --- | --- | --- | --- | --- | --- | --- | --- |
| Synaptophysin | Novocastra | Mouse | Monoclonal IgG1 | 27G12 | 1:100 | Long 30’ | H1 20’ |
| GFAP | Novocastra | Mouse | Monoclonal IgG1 | GA5 | 1:200 | Short | H1 20’ |
| Ki67 | Novocastra | Mouse | Monoclonal IgG1 | MM1 | 1:100 | Long 30’ | H2 40’ |
| p53 | Novocastra | Mouse | Monoclonal IgG2b | DO-7 | 1:100 | Short | H1 20’ |
| GAB1 | RabMAb | Rabbit | Monoclonal IgG1 | EPR375 | 1:600 | Short | H2 20’ |
| YAP1 | Santa Cruz Biotech | Mouse | Monoclonal IgG2a | sc-101199 | 1:300 | Long 30’ | H2 20’ |
| Filamin A | Thermo Fisher | Mouse | Monoclonal IgG1 | PM6/317 | 1:500 | Long 30’ | H1 20’ |
| Β-catenin | Biocare Medical | Mouse | Monoclonal IgG1 | CM 406A,C | 1:200 | Long 30’ | H1 40’ |

Supplementary table 1. Details and protocols of the 8 antibodies used in the experimental study using the Leica Bond III immuno-stainer. Dilutions were carried out using Bond Primary Antibody Diluent
