# Supplementary material for: Long-term outcome of the Milano-hyperfractionated accelerated radiotherapy strategy for high-risk medulloblastoma, including the impact of molecular subtype
Source: Neuro Oncol. 2024 Sep 27;27(1):209–18. doi: 10.1093/neuonc/noae189 (PMC11726337; doi:10.1093/neuonc/noae189)
Supplement: noae189_suppl_Supplementary_Table_S2 [file noae189_suppl_supplementary_table_s2.docx]

| **ID** | **M/F** | **AGE at diagnosis** | **posterior fossa syndrome yes/no** | **CSI Doses** | **Boost yes/no** | **HDCT yes/no, when** | **months of follow-up** | **IQ scores/School level attained/Job/Sport/ disabilities** | **Audiology** | **Endocrine alteration** |
| --- | --- | --- | --- | --- | --- | --- | --- | --- | --- | --- |
| 2 | M | 10.5 | no | 39 Gy | yes | no | 300 | University courses | normal | GH, Thyr |
| 5 | M | 18.5 | yes | 39 Gy | yes | no | 297 | University courses | loss 50 dB at 8000 Hz | GH, Thyr |
| 7 | M | 21.5 | yes | 39 Gy | yes | no | 284 | Job as engeneer | normal | nd |
| 10 | M | 10.8 | no | 39 Gy | yes | no | 282 | Secondary school/clerk | normal | met.sy, GH, Thyr, ACTH |
| 13 | M | 13.8 | yes | 39 Gy | yes | no | 276 | Not fulfilled secondary school program | normal | GH, testost |
| 16 | M | 32 | no | 39 Gy | yes | no | 271 | Lawyer, family and children | loss 20 dB at 8000 Hz | Thyr, testost |
| 17 | F | 20.6 | no | 39 Gy | yes | yes, post-CSI | 249 | Clerk part-time, depressed mood | loss 20 dB at 4000 Hz | GH, Thyr, ACTH, E/P |
| 20 | M | 7 | no | 39 Gy | no | yes, post-CSI | 260 | IQ 68 after 10 years, clerk, driving license | normal | GH, Thyr |
| 22 | M | 13 | yes | 39 Gy | yes | yes, post-CSI | 233 | Clerk, sport act. | hearing aids | GH, Thyr, Testost |
| 23 | M | 10.5 | yes | 39 Gy | yes | no | 239 | Secondary school with support | loss 60 dB at 8000 Hz | GH, Thyr |
| 24 | F | 26.5 | no | 39 Gy | yes | no | 93 | Married, one child | nd | nd |
| 28 | M | 13.7 | yes | 39 Gy | yes | no | 200 | University courses, driving license, sport act. | normal | GH, Thyr |
| 29 | F | 11.6 | no | 39 Gy | yes | no | 202 | nd | nd | nd |
| 31 | M | 15.3 | yes | 39 Gy | yes | yes, post-CSI | 172 | IQ 108 after 2 years, secondary school, clerk, depressed mood | normal | nd |
| 32 | F | 7.3 | yes | 39 Gy | no | yes, post-CSI | 194 | Completely dependent on parents, epiileptic | nd | GH, Thyr/ E/P |
| 33 | M | 7.1 | no | 39 Gy | yes | yes, post-CSI | 153 | Technical school, cardias stenosis | nd | GH, Thyr |
| 34 | M | 13.9 | no | 39 Gy | yes | no | 135 | Secondary school | nd | GH, Thyr |
| 36 | M | 11.4 | no | 39 Gy | yes | no | 168 | Barman, depressed mood | normal | Met Sy, Testost |
| 39 | M | 7.5 | no | 39 Gy | yes | yes, post-CSI | 153 | IQ 78 after 10 years, secondary school | loss 20 dB at 8000 Hz | GH, Thyr, Testost |
| 40 | M | 12.8 | no | 39 Gy | yes | yes, post-CSI | 155 | IQ 91 after 9 years, secondary school with support | bilateral hypoacusia | Met Sy, GH |
| 41 | M | 13.1 | no | 39 Gy | yes | yes, post-CSI | 154 | IQ 57 after 10 years, agricultural act., epileptic | normal | GH, Thyr |
| 47 | M | 5.5 | no | 39 Gy | yes | yes, post-CSI | 144 | Previous mental delay (IQ 45 after 10 years), secondary school with support | loss 20 dB at 8000 Hz | GH, Thyr, Testost |
| 54 | F | 16.8 | no | 39 Gy | no | yes, post-CSI | 114 | No job, sentimental engagement, radionecrosis at cerebellar peduncle operated after 5 years | light hypoacusia | E/P |
| 55 | M | 15.3 | no | 39 Gy | no | yes, post-CSI | 113 | University master courses, music player, social activities | loss 40 dB at 5000 Hx | GH |
| 57 | M | 12 | no | 39 Gy | yes | yes, post-CSI | 102 | Clerk, driving license | nd | GH, Thyr |
| 60 | F | 9.1 | no | 39 Gy | yes | no | 103 | IQ 77 after 6 years, technical school | loss 20 dB at 6000-8000 Hz | Met Sy, GH, E/P |
| 62 | M | 8 | no | 39 Gy | no | no | 89 | Secondary school with support, epileptic | normal | GH, Thyr |
| 63 | M | 15.4 | no | 39 Gy | no | yes, post-CSI | 88 | (previous mental delay and autism), voluntary job, sport act | normal | GH, Thyr, Testost, ACTH |
| 67 | F | 6.5 | no | 39 Gy | no | no | 79 | Secondary school with support, dancing | nd | GH, Thyr, E/P |
| 68 | M | 4.1 | no | 39 Gy | no | yes, pre-CSI | 80 | School with support | loss 20 dB at 8000 Hz | GH, Thyr |
| 79 | M | 20.3 | no | 39 Gy | yes | no | 52 | University courses, sport act. | loss 20 dB at 8000 Hz | GH, Thyr |
| 81 | F | 16.7 | no | 39 Gy | yes | no | 52 | Teacher at nursery school | nd | GH, Thyr; E/P |
| 84 | F | 10.5 | no | 39 Gy | yes | no | 46 | School with support | normal | Met Sy, GH |
| 86 | M | 18.1 | no | 39 Gy | yes | no | 39 | University courses | normal | Thyr |
| 89 | M | 8.3 | no | 39 Gy | yes | no | 27 | School with support | normal | GH |
|  |  |  |  |  |  |  |  |  |  |  |
| 1 | M | 6.6 | no | 31.2 Gy | yes | no | 263 | Secondary school | loss 20 dB at 8000 Hz | GH, Thyr |
| 6 | M | 7.1 | no | 31.2 Gy | yes | no | 289 | University degree, competitive biking | loss 20 dB at 8000 Hz | GH, Thyr |
| 8 | F | 7.5 | no | 31.2 Gy | yes | no | 290 | Tailored school program, parents refused rehabilit. | nd | GH |
| 9 | M | 3.3 | no | 31.2 Gy | yes | yes, post-CSI | 273 | Still ongoing rehabilitation, living with parents, late radionecrosis in cerebellar peduncle | coclear-labirynthic disfunction | GH |
| 15 | F | 6.8 | no | 31.2 Gy | yes | yes, post-CSI | 271 | Epileptic, amaurotic | severe hypoachusia | Met Sy, GH, Thyr |
| 35 | F | 4.6 | no | 31.2 Gy | yes | yes, post-CSI | 177 | IQ 90 after 2 years | nd | GH, Thyr |
| 37 | M | 6.3 | no | 31.2 Gy | yes | no | 173 | IQ 77 after 8 years, Secondary school, animal care | loss 20 dB at 8000 Hz | GH, Thyr |
| 38 | F | 4.0 | no | 31.2 Gy | yes | yes, post-CSI | 170 | IQ 77 after 10 years, social job in a community, dance | nd | GH, Thyr, E/P |
| 46 | F | 7.1 | no | 31.2 Gy | yes | yes, post-CSI | 128 | IQ 85 after 5 years, scholastic support | hearing aids | GH, Thyr, E/P |
| 51 | M | 5.9 | no | 31.2 Gy | yes | yes, post-CSI | 137 | IQ 83 after 10 years, secondary school, active social life | loss 20 dB at 8000 Hz | GH, Thyr |
| 52 | M | 9.11 | no | 31.2 Gy | yes | yes, post-CSI | 129 | Tailored school program | nd | GH, Thyr |
| 53 | M | 9.11 | no | 31.2 Gy | yes | yes, post-CSI | 125 | Hemiparesis, cataract | severe hypoacusia | GH |
| 58 | F | 7.5 | no | 31.2 Gy | yes | no | 109 | IQ 106 after 2 years, secondary school, sport act. | nd | GH, Thyr |
| 64 | F | 4.7 | no | 31.2 Gy | yes | no | 82 | IQ 104 after 2 years, tailored school program | normal | GH, Thyr |
| 71 | M | 4.1 | no | 31.2 Gy | yes | sì | 72 | IQ 103 after 6 years | normal | Thyr |
| 72 | M | 9.11 | no | 31.2 Gy | yes | sì | 66 | Secondary school without support, transient bulbar ischemia | loss 20 dB at 6000-8000 Hz | GH, Thyr, testost |
| 73 | F | 8.9 | no | 31.2 Gy | yes | no | 67 | Secondary school without support | normal | Thyr |
| 75 | M | 5.0 | no | 31.2 Gy | yes | yes, pre-CSI | 65 | IQ 88 after 4 years, normal school program, sport act. | nd | GH, Thyr |
| 77 | M | 4.2 | no | 31.2 Gy | yes | yes, pre-CSI | 57 | Previous mental delay | nd | GH |
| 78 | M | 4.7 | no | 31.2 Gy | yes | no | 52 | IQ 94 after 2 years, school with support | hearing aids | GH, Thyr |
| 80 | F | 7.2 | no | 31.2 Gy | yes | no | 54 | IQ 88 after 4 years, school with support, sport act. | loss 20 dB at 6000-8000 Hz | GH, Thyr |
| 82 | F | 5.7 | no | 31.2 Gy | yes | no | 58 | school with support, active social life | loss 20 dB at 8000 Hz | GH, Thyr, precocious puberty |
| 83 | M | 6.7 | no | 31.2 Gy | yes | yes, pre-CSI | 52 | IQ 84 after 2 years, school with support, sport act. | normal | GH, Thyr |

**Supplemental Table 2.**

Legenda: IQ = intelligence quotient; sport act .= sport performed with continuity i.e. at least twice a week, Met. Sy = metabolic syndrome; GH =

Growth Hormone deficit, Thyr = hypothyroidism; testost = hypogonadism hypogonadotropic in males; E/P = estroprogestinic deficit
