# Supplementary figures and images for: Long-term outcome of the Milano-hyperfractionated accelerated radiotherapy strategy for high-risk medulloblastoma, including the impact of molecular subtype
Source: Neuro Oncol. 2024 Sep 27;27(1):209–18. doi: 10.1093/neuonc/noae189 (PMC11726337; doi:10.1093/neuonc/noae189)

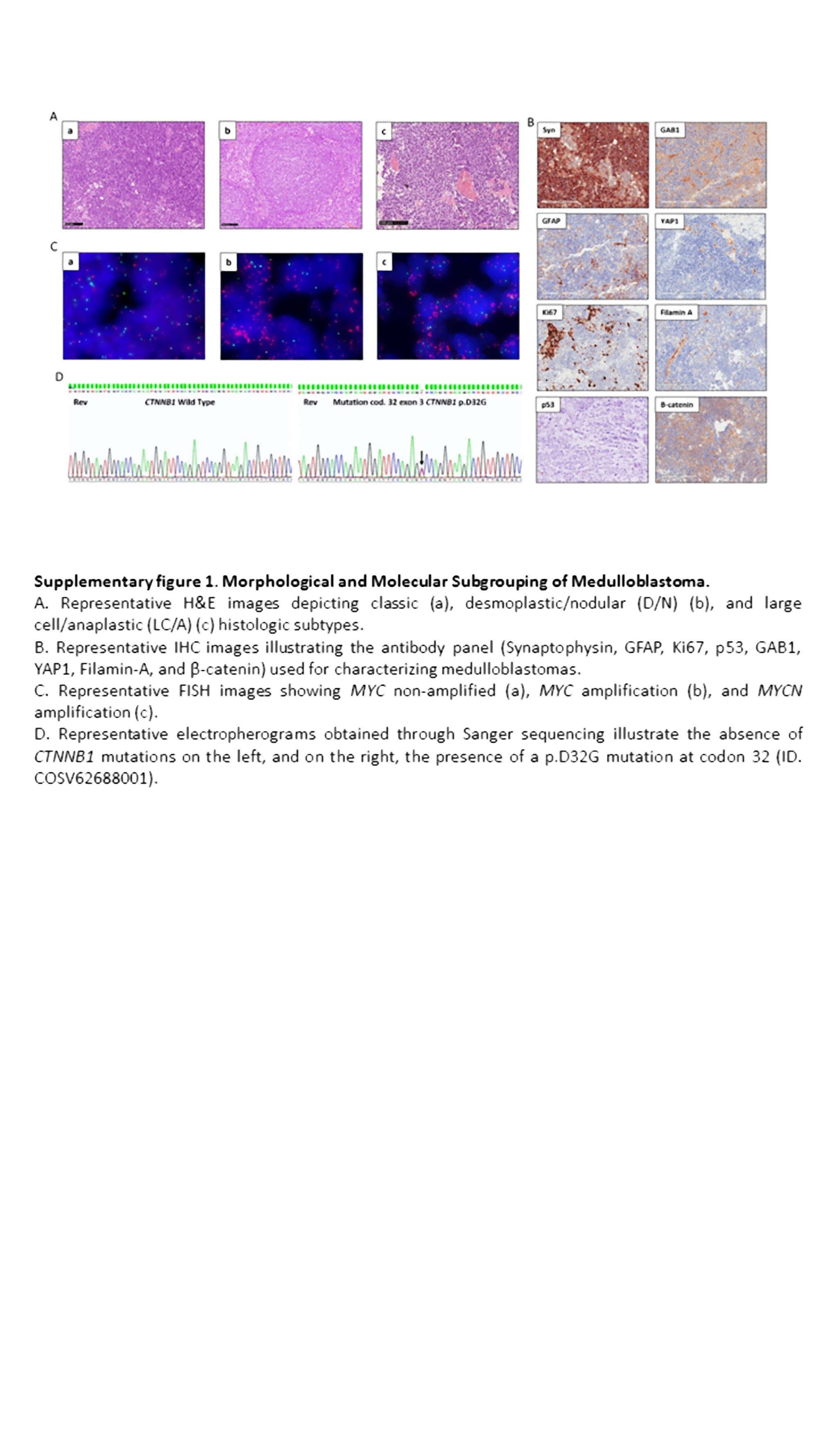

Supplement: noae189_suppl_Supplementary_Figure_S1 [file noae189_suppl_supplementary_figure_s1.jpeg]

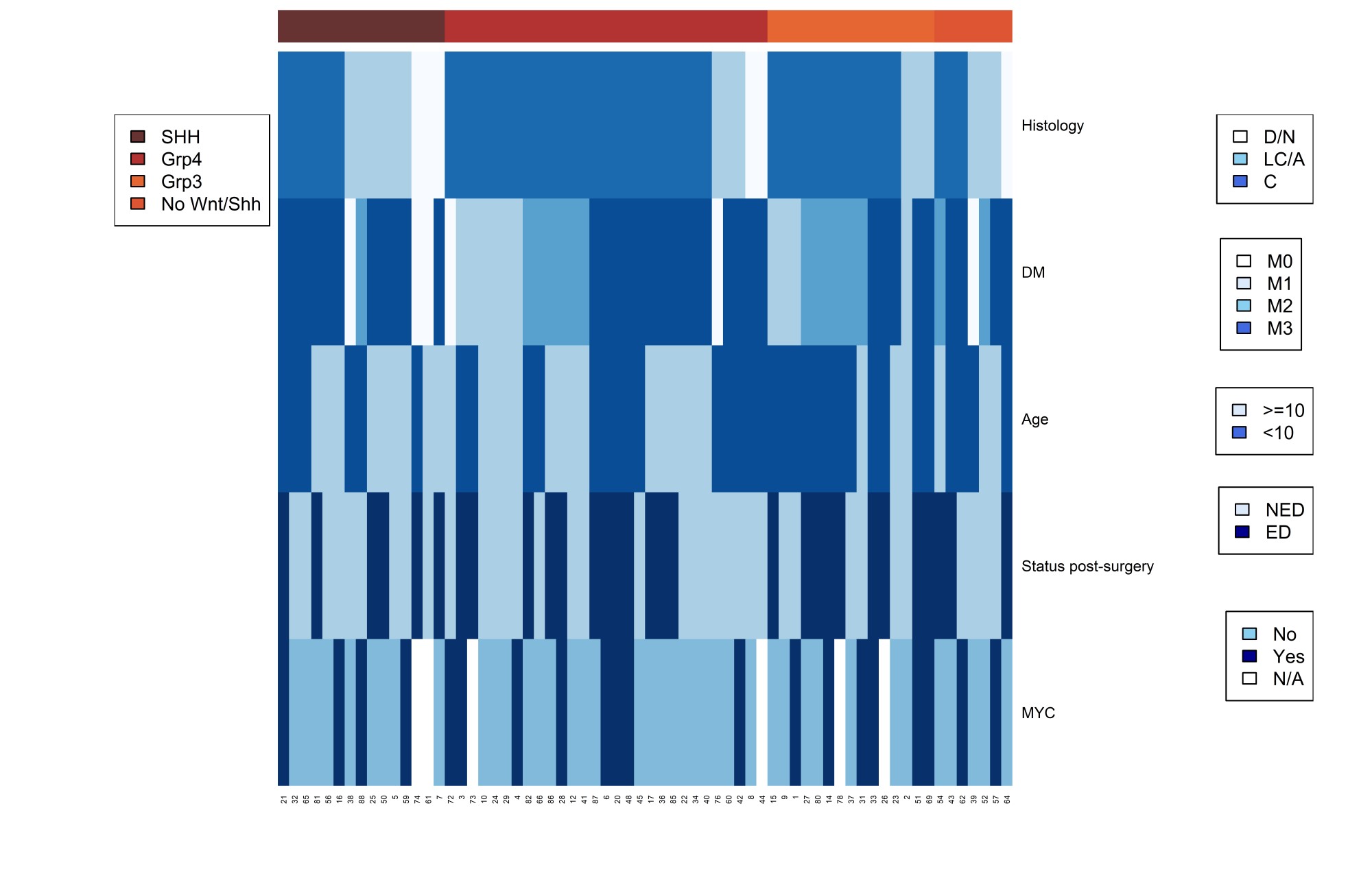

Supplement: noae189_suppl_Supplementary_Figure_S2 [file noae189_suppl_supplementary_figure_s2.jpeg]
