# Supplementary material for: Long-term outcome of the Milano-hyperfractionated accelerated radiotherapy strategy for high-risk medulloblastoma, including the impact of molecular subtype
Source: Neuro Oncol. 2024 Sep 27;27(1):209–18. doi: 10.1093/neuonc/noae189 (PMC11726337; doi:10.1093/neuonc/noae189)
Supplement: noae189_suppl_Supplementary_Material [file noae189_suppl_supplementary_material.docx]

**Morphological subgrouping**

All immunohistochemistry (IHC) tests were conducted in a clinically-accredited laboratory, the Neuropathology Unit at Sapienza University of Rome, responsible of Italy's national program for the centralization of pediatric brain tumors. The morphological assessment of medulloblastomas relied on Hematoxylin-Eosin (H&E) and reticulin staining. Through this evaluation, three histologic subgroups were distinguished: classic medulloblastma (MB), the most prevalent subtype, desmoplastic/nodular (D/N) MB, and large cell/anaplastic (LC/A) MB. Microscopically, the classic MB shows clusters of small-round neoplastic cells exhibiting densely packed undifferentiated embryonal characteristics, marked by the presence of mitotic figures and apoptotic bodies. Additionally, Homer Wright rosettes can be discernible. The D/N MB displays densely packed undifferentiated cells featuring hyperchromatic and pleomorphic nuclei. This subtype generates a dense intercellular reticulin fibre network, with identifiable nodular areas lacking reticulin. The LC/A MB is characterized by anaplasia and pronounced nuclear pleomorphism, a high mitotic rate, and the presence of apoptotic bodies. Nuclear moulding and cell wrapping can also be found in this subtype (Supplementary figure 1A). For the diagnosis of LC/A MB, a severely anaplastic or a large cell component or a mixture of both should be predominant (more that 50% of the tumor area).

Molecular subgroups were primarily assessed using IHC. IHC procedures were conducted employing a streptavidin–biotin–immunoperoxidase protocol on an automated immuno-stainer (Leica Bond III), following the manufacturer’s instructions. Specific conditions were applied for each primary antibody incubation of the panel (Synaptophysin, GFAP, Ki67, p53, GAB1, YAP1, Filamin-A, and β-catenin). Details are available in Supplementary table 1.

In most MBs, neoplastic cells exhibited diffuse positivity for Synaptophysin, with only focal positivity for GFAP. The Ki67 labelling index ranged between 20-70%. The nuclear expression of p53, when detected, suggests the presence of mutations in the TP53 gene. The IHC panel involving GAB1, YAP1, Filamin A, and nuclear β-catenin aided in classifying MBs into Sonic Hedgehog (SHH), WNT, or non-SHH/non WNT groups. SHH medulloblastoma was identified by triple positivity for GAB1, YAP1, and Filamin A. WNT MB was characterized by nuclear staining for β-catenin, confirmed by *CTNNB1* mutations on exon 3. The non-SHH/non-WNT group lacked triple positivity for GAB1, YAP1, and Filamin A, as well as nuclear staining for β-catenin (Supplementary figure 1B). Cases with unclear nuclear β-catenin staining underwent analysis via Sanger sequencing. Genomic DNA was purified using the DNA Blood & Tissue Kit (Qiagen) according to the manufacturer's guidelines. Sanger direct sequencing was performed on purified PCR-product of 400 bp, spanning the mutations cluster region in exon 3 of *CTNNB1* (Supplementary figure 1D, specific details available upon request).

Additionally, samples were examined via Fluorescence in situ Hybridization (FISH) to detect *MYC* and *MYCN* amplification. FISH has a high sensitivity in detecting amplification, allowing the evaluation of gene copy numbers at a single-cell level and correlation with the morphologic features of neoplastic tissues. Sections of five-micron thickness were obtained from FFPE samples; slides were de-paraffinized, treated with pretreatment solution (1 mol/L sodium thiocyanate), and immersed in pepsin solution (0.65% in protease buffer). *MYC* and *MYCN* copy number variations were assessed using *MYC* (8q24.21) Orange/CEP8 Green and *MYCN* (2p24.3) Orange/CEP2 Green probes, following the manufacturer's protocol (Empire Genomics). Sections were counterstained with DAPI and examined using an Axio Imager M1 microscope (Carl Zeiss). Signal counts were conducted in selected neoplastic areas, evaluating at least 200 nuclei for each sample. A Locus-Specific/CEP signal ratio between 1-2 indicated gain, while a signal ratio >2 indicated amplification, with a cut-off value of >10% neoplastic cells (Supplementary figure 1C).The MYC/MYCN gene amplification was reported when a minimum of 9 locus-probe signals compared to 2 reference-probe signals were found in more than 10% of tumor cells. In some sample, locus oncogene signals could also appear as innumerable or numerous clusters.
